# Identification of optimal thalassemia screening strategies for migrant populations in Thailand using a qualitative approach

**DOI:** 10.1186/s12889-021-11831-4

**Published:** 2021-10-06

**Authors:** Julia Z. Xu, Meghan Foe, Wilaslak Tanongsaksakul, Thidarat Suksangpleng, Supachai Ekwattanakit, Suchada Riolueang, Marilyn J. Telen, Bonnie N. Kaiser, Vip Viprakasit

**Affiliations:** 1 Department of Medicine, Duke University, Durham, USA; 2 National Heart, Lung, and Blood Institute, National Institutes of Health, 10 Center Drive Room 6N240C, Bethesda, MD 20892 USA; 3 Department of Hematology/Oncology, University of California San Francisco Benioff Children’s Hospital Oakland, Oakland, USA; 4 Department of Pediatrics, Laem Chabang Hospital, Laem Chabang, Thailand; 5 Siriraj-Thalassemia Center, Mahidol University, Bangkok, Thailand; 6 Duke Global Health Institute, Duke University, Durham, USA; 7 Department of Anthropology and Global Health Program, University of California San Diego, La Jolla, USA; 8 Department of Pediatrics, Faculty of Medicine Siriraj Hospital, Mahidol University, 2 Wanglang Road, Bangkoknoi, Bangkok, 10700 Thailand

**Keywords:** Thalassemia, Migrants, Barriers to care, Healthcare access, Migration policy, Prenatal screening, Genetic testing, Termination of pregnancy, Thailand, Southeast Asia

## Abstract

**Background:**

Thalassemia is a common inherited hemoglobin disorder in Southeast Asia. Severe thalassemia can lead to significant morbidity for patients and economic strain for under-resourced health systems. Thailand’s thalassemia prevention and control program has successfully utilized prenatal screening and diagnosis to reduce the incidence of severe thalassemia in Thai populations, but migrant populations are excluded despite having high thalassemia prevalence. We sought to identify key barriers to and facilitators of thalassemia screening and to develop tailored recommendations for providing migrants with access to thalassemia prevention and control.

**Methods:**

We conducted 28 in-depth interviews and 4 focus group discussions (FGDs) in Chonburi, Thailand with Myanmar and Cambodian migrants, Thai healthcare providers, Thai parents of children affected by thalassemia, and migrant agents.

**Results:**

Participant narratives revealed that migrants’ lack of knowledge about the prevalence, manifestations, severity, and inherited nature of thalassemia led to misconceptions, fear, or indifference toward thalassemia and screening. Negative perceptions of pregnancy termination were based in religious beliefs but compounded by other sociocultural factors, presenting a key obstacle to migrant uptake of prenatal screening. Additionally, structural barriers included legal status, competing work demands, lack of health insurance, and language barriers. Participants recommended delivering public thalassemia education in migrants’ native languages, implementing carrier screening, and offering thalassemia screening in convenient settings.

**Conclusions:**

An effective thalassemia prevention and control program should offer migrants targeted thalassemia education and outreach, universal coverage for thalassemia screening and prenatal care, and options for carrier screening, providing a comprehensive strategy for reducing the incidence of severe thalassemia in Thailand and establishing an inclusive model for regional thalassemia prevention and control.

## Background

Thalassemia syndromes are common inherited disorders characterized by mutations leading to the underproduction of α- or β-globin proteins and are a major contributor to the global burden of anemia [1]. Thalassemia carriers are typically asymptomatic but can have severely affected children due to the autosomal recessive nature of the disease, highlighting the importance of thalassemia screening. Worldwide, an estimated 56,000 births are affected annually by severe forms of thalassemia (i.e. hemoglobin Bart’s hydrops fetalis, β-thalassemia major, and hemoglobin E/β-thalassemia) [2] that can lead to complications such as chronic transfusion dependent anemia, splenomegaly, bone deformities, perinatal death, and life-threatening maternal obstetric complications [3].

Thailand, like surrounding Southeast Asian (SEA) nations, has one of the highest burdens of thalassemia globally, with ~ 1 in 100 births affected by severe thalassemia [4, 5]. As a result of its relative stable and prosperous economy, Thailand has become a main destination for regional migration, hosting ~ 3.9 million migrants, mainly from neighboring Myanmar, Cambodia, and Laos [6]. These large-scale migrations have the potential to change the epidemiology of thalassemia significantly, representing new challenges to health systems in impacted countries [7–10]. However, little attention has been paid to mass migrations within SEA and its implications on regional prevention and control of thalassemia.

### Approach to thalassemia prevention and control in Thailand

Thailand is one of several countries with a successful national thalassemia prevention and control program [11–14] and has observed a decreased incidence of births affected by hydrops fetalis and severe β-thalassemia in some regions [15–17]. Programs typically employ a combination of widespread public education and awareness efforts, carrier or preconception screening, genetic counseling, prenatal screening (PNS) and diagnosis (PND), and preimplantation diagnosis [11]. The choice of screening strategy can be driven by cultural and religious acceptance; for example, in Middle Eastern countries such as Iran, premarital thalassemia screening is required due to the religious and legal prohibition of termination of pregnancies affected by thalassemia [18].

Thailand’s program focuses on public education, PNS/PND, and genetic counseling. Antenatal care (ANC) is central to Thailand’s strategy, as thalassemia screening and education are typically performed during ANC visits. Pregnant women found to carry a thalassemia mutation are asked to bring in their partners for PNS, and at-risk couples are referred for PND [16, 17]. In cases where the fetus is identified to be at high risk of severe thalassemia, a qualified provider must then document the pregnant woman as having physical or mental health reasons necessitating pregnancy termination before the procedure is offered, as elective abortion remains illegal in Thailand [19]. In addition, access to legal abortion care is greatly affected by local hospital and provider interpretations of abortion laws, as well as the economic and legal status of the patient [19].

### Challenges for thalassemia screening in migrant populations

Migrant populations face unique barriers to thalassemia prevention, which may undermine the success of the current Thai prevention and control program. For instance, the Thai government provides both basic universal health coverage and specific coverage for thalassemia prevention procedures to Thai citizens but not to migrants. Other studies have cited insufficient knowledge of thalassemia among migrant populations as barriers to thalassemia prevention efforts [20–22]. We conducted a knowledge, attitudes, and practice (KAP) survey among Thai individuals and Myanmar and Cambodian migrant workers in Chonburi, Thailand and found a startling lack of awareness of thalassemia among migrants compared to Thai (4% vs. 80% awareness, respectively) [23]. Ours and previous studies suggest that the lack of awareness about thalassemia among migrant populations may be related to a lack of public thalassemia education for migrants [24, 25].

Migrants may also hold cultural values and attitudes toward blood disorders, inherited diseases, prenatal testing, and termination of pregnancy that differ from those of the host country and may affect uptake of thalassemia screening [20, 21]. Without concerted efforts to address these differences, high-income countries have faced difficulties developing effective thalassemia prevention and control strategies for migrant communities [7]. The limited literature on thalassemia in SEA migrant communities suggests potential barriers to thalassemia screening related to conflicting values between biomedicine and preferences such as traditional medicine, religious opposition to abortion, and a desire to have more children as a measure of prosperity [21, 26]. The vulnerability of migrant populations additionally poses ethical challenges to thalassemia screening due to the potential risks of exacerbating social isolation or psychosocial stress in migrants diagnosed with thalassemia carrier status and of providers exerting undue pressure on migrants to be screened [21, 26].

Additionally, migrants face structural barriers to accessing healthcare in general. In line with recent efforts by the Thai government to strengthen Thailand’s industrial sector by incentivizing intra-regional migration and legalizing the migrant workforce, a migrant health insurance scheme was established. Yet this insurance scheme is vastly underutilized due to complicated legal requirements and registration procedures, high costs, and reluctance of hospitals to facilitate the registration process [27, 28]. Migrant agents, often bilingual members of the migrant community, are hired by employers and migrants to assist with this process, but their services are not accessible to all migrants [28]. Even among migrants with health insurance, healthcare utilization may remain low due to a constellation of structural barriers, including high out-of-pocket expenditures, lack of transportation, long wait times, language barriers, constructs of health or illness which differ from the medical establishment, low health literacy, lack of social support, fear among undocumented migrants of being arrested, and unfriendly or discriminatory health worker attitudes toward migrants [27–32].

Despite these barriers, previous literature has found a high rate of utilization of healthcare services such as ANC and postpartum care among migrants in Thailand, [33] particularly when services were affordable, conveniently located, and provided by friendly health workers [31]. Similarly, despite identifying negative attitudes toward abortion among Myanmar and Cambodian subjects, we found the uptake of thalassemia screening to be 97% in these cohorts [23]. These counterintuitive findings suggest that thalassemia screening is feasible for migrant populations when the approach accommodates the knowledge, cultural values, and structural barriers faced by migrants. Therefore, we sought to facilitate future thalassemia screening efforts in migrants by characterizing barriers and facilitators to screening using qualitative methods. Through in-depth interviews and focus group discussions (FGDs) with a variety of stakeholders, including Myanmar and Cambodian migrant workers, migrant agents, Thai healthcare providers, and Thai parents of children with severe thalassemia, we aimed to identify optimal strategies for thalassemia screening in SEA migrant populations.

## Methods

We conducted a mixed-methods study of adult migrants and Thai citizens residing in Chonburi, Thailand and report here the qualitative piece. Chonburi is an industrial province with one of the largest migrant populations in Thailand. Laem Chabang Hospital (LCH) was chosen as the recruitment site because it serves the majority of migrants in the port city of Laem Chabang, Chonburi, where ~ 200,000 migrant workers reside. LCH is a public secondary care center with 90 beds and 20 physicians operating under the Ministry of Public Health and provides health registration services, a prerequisite for migrants to obtain legal documentation. As some migrants were presenting for health registration for the first time, we had an opportunity to access an unregistered, or undocumented, migrant population that is otherwise challenging to enroll. As such, health registration-based studies provide a particularly efficient means of sampling both documented and undocumented migrants.

The goal of our qualitative study was to obtain a rich understanding of lay migrants’ knowledge and attitudes surrounding thalassemia, but as demonstrated in our previous work, [23] lay migrant workers have extremely low levels of awareness of thalassemia. To ensure sufficiently rich data, we recruited migrant workers as well as Thai healthcare providers (defined as doctors or nurses from LCH) and migrant agents (or agents, defined as individuals paid to help to facilitate employment and immigration procedures for migrant clients) who interact frequently with migrants in the healthcare setting. As a comparison group, we also recruited Thai parents of children with thalassemia. In-depth interviews were performed with migrant workers, providers, and agents, while FGDs (conducted in mixed-gender groups of 5) were performed with 2 groups of providers and 2 groups of Thai parents. All participants were recruited through a combination of purposive sampling and participant referrals from various outpatient settings at LCH (Table 1). Participants were selected if they had either pre-existing knowledge of thalassemia (migrant workers and Thai parents) or experience working with migrants receiving healthcare in Thailand (agents and providers).

**Table 1:** Summary of recruitment populations and methods for in-depth interviews and focus group discussions

Research Method	Study Population	Nationality of Participants	Sample Size	Setting of Recruitment	Sampling Method
**In-depth Interviews**	Migrant workers	Myanmar, Cambodian	9	Presenting for outpatient or health registration services at Laem Chabang Hospital (LCH)	Purposive sampling; provider and migrant referrals
	Migrant agents	Myanmar, Cambodian, Thai	9	Presenting for work at LCH	Purposive sampling; provider and agent referrals
	Providers	Thai	10	Presenting for work at LCH	Purposive sampling; provider referrals
**Focus Group Discussions (FGDs)**	Providers	Thai	2 groups of 5 participants	Presenting for work at LCH	Purposive sampling; provider referrals
	Parents of children with thalassemia	Thai	2 groups of 5 participants	Presenting for outpatient services at LCH	Provider referrals

Semi-structured interview and FGD guides were developed covering the following topics: lay migrants’ knowledge of thalassemia disease, thalassemia carriers, and inherited disease, with comparisons to lay Thai citizens; lay migrants’ perceptions of thalassemia, blood disorders, termination of pregnancy, medical care in Thailand, and alternative sources of healthcare, with comparisons to lay Thai citizens; and lay migrants’ experience of healthcare and the role of language, health literacy, economic status, legal status, health registration, health insurance, employment, and transportation in accessing healthcare. Of note, the knowledge of providers themselves was not studied or compared to the knowledge of lay individuals; rather, these professionals served as information-rich sources and provided their perceptions of both lay migrants’ and lay Thai citizens’ knowledge of and attitudes surrounding thalassemia. In our sample, all providers and parents were Thai in nationality, while agents were mostly Myanmar or Cambodian (with only one agent being Thai). Thus, agents provided an interesting dual perspective, as professionals who regardless of their nationality shared their experience of working with the lay migrant population, as well as members of the migrant community who provided insights into sociocultural factors affecting thalassemia screening and healthcare utilization.

Interviews and FGDs were conducted in Thai or English, with interpreters as necessary. All sessions were audio-recorded and conducted in a private setting. Recordings were transcribed and translated from Thai into English by either bilingual native Thai speakers or a professional translation service. A notetaker was present at all FGDs and provided a written account of situational data. Transcripts were imported into MAXQDA software (Version 2018, VERBI GmbH, Berlin, Germany) for analysis. Thematic analysis [34] was used to develop a conceptual framework of factors influencing the feasibility and acceptability of thalassemia prevention among migrants in Thailand.

The codebook included inductive codes identified from the text, as well as deductive codes based on themes identified from the preceding KAP survey [23]. After reading and memoing transcripts, coding was performed by two coders, with intercoder reliability assessed for the first one-third of transcripts to harmonize coding strategies and iteratively develop the codebook. All transcripts were then coded using the finalized codebook. Analysis was guided by explanatory questions derived from the KAP survey, as well as an overarching goal of categorizing the various barriers and facilitators into main themes (Table 2).

**Table 2:** KAP survey findings, corresponding explanatory questions, and main themes and related recommendations

KAP Survey Finding	Explanatory Question	Major Theme	Recommendations
• Only 4% of migrants reported awareness of thalassemia, vs 80% of Thai.	• What do migrants know about thalassemia?	Migrants lack awareness and knowledge about thalassemia, leading to negative attitudes toward thalassemia carriers and thalassemia screening.	An optimal initial strategy includes implementation of targeted education, delivered in migrants’ native languages through antenatal care (ANC), public education, or mass media.
• 45% of migrants and 33% of Thai believed thalassemia to be an infectious disease.	• How do migrants learn about thalassemia?		
• 64% of migrants (vs. 57% of Thai) believed that thalassemia carriers require blood transfusions.	• What do migrants think about people with thalassemia?		
• 91% of migrants (vs. 54% of Thai) believed that carriers can develop thalassemia major.	• What do providers think migrants know or feel about thalassemia?		
• Migrants more “strongly disagreed” with termination of pregnancy compared to Thai.	• What do migrants think about the prevention of thalassemia in general?	Sociocultural factors influence uptake of thalassemia screening and termination of pregnancy for migrants.	Carrier screening may be more effective than prenatal screening for thalassemia prevention in migrant populations.
• Migrants felt strongly that they would not prevent the birth of a fetus affected by thalassemia.	• What do migrants think about termination of pregnancy?		
• One-quarter of migrants surveyed were unregistered (likely undocumented or irregular).	• What systemic barriers or facilitators of thalassemia screening exist for migrants?	Structural and systemic factors affect migrants’ access to thalassemia screening.	Increasing migrants’ access to legal documentation and health insurance, providers’ access to interpreter services and cultural competency training, and conducting thalassemia screening in convenient settings for migrants will aid thalassemia prevention and control efforts.
• Migrants reported few doctor’s visits and low rates of health insurance utilization.	• What do Thai think about the prevention of thalassemia in migrants?		

## Results

Of the 28 in-depth interview participants, 53% were female, and all 3 nationalities were represented (Table 3). Among sub-samples, Thai providers were oldest (mean age 43 years), and migrant workers were youngest (mean age 28 years). Migrants reported residing in Thailand for 8.2 years on average, with migrant agents reporting longer lengths of residence (mean 9.2 years, range 5–25 years) and possessing greater Thai fluency and more familiarity with Thai health systems compared to migrant workers (mean 7.2 years, range 4–10 years). FGD participants were Thai and predominantly female, with only 2 male parents represented.

**Table 3:** Demographic information for participants in in-depth interviews and focus group discussions (FGDs)

	Interviews				FGDs*		
	Migrants ***N*** = 9	Agents *N* = 9	Providers ***N*** = 10	Total ***N*** = 28	Provider *N* = 10	Parent *N* = 10	Total ***N*** = 20
**Female, (%)**	4 (44%)	3 (33%)	8 (80%)	15 (53.6%)	10 (100%)	8 (80%)	18 (90%)
**Mean age, years (range)**	28 (18–36)	36 (26–54)	43 (32–53)	36 (18–54)	32.6 (23–52)	35.5 (20–45)	34.1 (20–52)
**Country of origin**							
Thailand, (%)	0	1	10	11 (39.3%)	10 (100%)	10 (100%)	20 (100%)
Myanmar, (%)	4	2	0	6 (21.4%)	0	0	0
Cambodian, (%)	5	6	0	11 (39.3%)	0	0	0

*There were 4 FGDs conducted; these demographics reflect individual participants

**Fig. 1:**
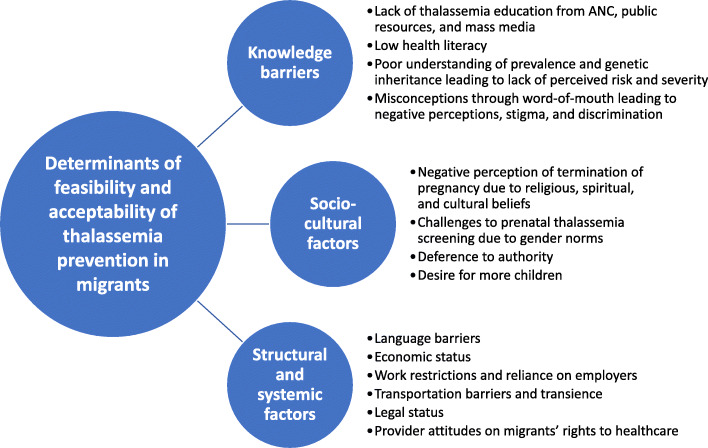
Barriers and facilitators affecting feasibility and acceptability of thalassemia prevention in migrant populations in Thailand

We identified three main themes reflecting different categories of barriers and facilitators to successful thalassemia prevention in migrants (Fig. 1): (1) lack of knowledge about thalassemia leading to negative attitudes toward screening, (2) sociocultural factors influencing uptake of thalassemia screening and termination of pregnancy, and (3) structural and systemic factors affecting access to thalassemia screening and interventions. These factors inform the feasibility and acceptability of the existing system of thalassemia prevention and control measures for migrant vs Thai populations and provide context for the discussion of recommendations of optimal or alternative approaches to thalassemia prevention for migrants.

### Knowledge barriers

Participant narratives supported previous findings that migrants have extremely low levels of thalassemia awareness and knowledge compared to Thai. Only a handful of migrants had knowledge of thalassemia beyond having “heard this word”, and some migrants equated thalassemia with anemia or could “describe the symptoms [of thalassemia], but they don’t know the name of the disease” (A04, Thai female agent, 41-year-old [41yo]). Narratives also illustrated that migrants had little to no knowledge of the hereditary nature and reproductive implications of thalassemia, or of the general concept of genetic disease. In addition, Thai participants, but not migrants, recognized thalassemia as a prevalent illness in SEA. On the other hand, Thai providers and parents acknowledged a similar lack of knowledge about thalassemia among the general Thai public. A few Thai providers felt that both migrants’ and Thai individuals’ level of knowledge about thalassemia were related to their level of educational attainment.

Of the few migrants who felt knowledgeable about thalassemia, nearly all had heard about it through word-of-mouth from friends, family, coworkers, or acquaintances, highlighting the importance of learning about thalassemia through personal experience and connections. Yet participants provided clear evidence that these informal avenues of thalassemia education are vulnerable to misinformation, with several migrants holding misconceptions about thalassemia being an infectious disease or nutritional deficiency. One migrant worker reported learning from her coworkers that she should not share water containers with an affected individual due to the contagious nature of thalassemia (M02, Myanmar female migrant, 32yo). Similarly, a migrant agent and landlord enthusiastically discussed how she frequently educated her migrant tenants and clients on the prevention of thalassemia through use of condoms, comparing it to prevention of HIV (A04, Thai female agent, 41yo).

#### Antenatal care as an entry point for thalassemia awareness and education

In contrast to migrants, Thai provider and parent narratives suggest that the majority of Thai people learn about thalassemia through antenatal care (ANC) clinics, where they receive formalized face-to-face counseling about thalassemia prevalence, symptoms, severity, and reproductive implications. Providers reported offering extensive prenatal counseling on thalassemia to their patients during ANC, and the majority of Thai parents confirmed this experience. Yet nearly all migrant participants reported that they were not told by providers about thalassemia during ANC visits, or that they were insufficiently informed about the severity of the disease. When asked about this perceived discrepancy, several providers reported providing equitable thalassemia education to migrants but acknowledged significant language and communication barriers. A few providers expressed frustrations about educating migrants about thalassemia, feeling that migrants “won’t understand anything” and that educating them about thalassemia was a futile effort, while others felt that it was possible but time-consuming to explain thalassemia to migrants. Additionally, there was a pervasive sentiment among providers that migrants were unquestioningly obedient compared to Thai and therefore may not require extensive education or counseling about thalassemia because they would ultimately defer to the providers’ recommendations.

#### Alternative strategies for thalassemia education

Participants suggested several strategies for thalassemia education tailored to migrants’ educational and communication needs – for instance, the utilization of visual aids and illustrations to explain patterns of thalassemia inheritance, or holding separate ANC clinics for migrants, during which interpreters would be present to help providers overcome language barriers. In particular, providers felt that the lack of written educational materials in migrants’ native languages was a hinderance.

Alternatively, many participants felt that thalassemia education for migrant and Thai populations alike should be delivered outside of the ANC setting through mass media. One provider believed that the thalassemia knowledge gap between Thai and migrants was largely attributable to differential access to mass media. Indeed, the majority of Thai parents, as well as some migrant agents, reported hearing about thalassemia through media outlets, such as news, television shows, or websites. In contrast, no migrant workers mentioned these avenues of learning about thalassemia. Participants agreed that widespread public education efforts through mass media, delivered in migrants’ native languages, would be essential to disseminate knowledge and increase uptake of thalassemia screening among migrant communities. Thai participants added that the government could provide further thalassemia outreach and education programs to rural villages and industrial companies employing migrant workers.

#### Perceived health and lack of thalassemia exposure drive migrant attitudes toward thalassemia screening

Participants felt that migrants’ self-perceptions of health and their lack of exposure to individuals affected by thalassemia resulted in indifference or apathy about thalassemia screening. Many migrant workers reported that they were rarely sick and did not think they could be affected by thalassemia. Participants also suggested that migrant workers may be healthier than the general population, given that immigration eligibility was often conditional upon health status. This perception of health may lead migrants to view preventative health measures such as thalassemia screening as low priority.

Thai parents’ narratives also pointed to a lack of personal experience with serious complications of thalassemia as a predominant reason for migrants’ perceived indifference. Thai parents pointed to their own indifference towards and perceived lack of risk of thalassemia prior to their child’s diagnosis of severe thalassemia. Though this study did not capture narratives of migrant parents of children with thalassemia, one migrant subject had a child suspected to have thalassemia and expressed great concern about thalassemia as a “serious and scary disease” (M07, Cambodian female migrant, 24yo).

Migrant narratives further revealed that misconceptions about thalassemia severity and inheritance can produce negative attitudes toward thalassemia screening. Some migrants expressed the desire to avoid carrier screening out of fear of being diagnosed with a severe disease, while others expressed concerns that employers could discriminate against, fire, or even deport individuals diagnosed with thalassemia. In comparison, Thai parents did not express concern for the diagnosis of thalassemia, but rather for the incidental finding of another serious illness, like cancer or diabetes. Finally, participant narratives demonstrated that misconceptions about thalassemia can result in discrimination and social isolation. One participant described her interaction with a coworker rumored to have thalassemia, saying, “I don’t usually talk to her because I’m afraid what she has will be contagious” (M02, Myanmar female migrant, 32yo). These findings show that knowledge and attitudes about thalassemia are linked, and that poor knowledge of thalassemia can be a substantial barrier to the acceptability of thalassemia screening in migrant populations.

### Sociocultural factors

Participant narratives illustrated that gender roles and attitudes toward termination of pregnancy can impact health seeking and reproductive behaviors, as well as perceptions of thalassemia prevention. Both male and female Thai parents viewed thalassemia screening as more of a concern for women than men, stating that it was the woman’s responsibility to “find husbands who aren’t affected by the disease” (FGD02, male Thai parent). Though Thai mothers generally believed that male partners should undergo thalassemia screening, when recounting their own experiences with a male partner who failed to present for PNS, they were hesitant to blame and even offered excuses for their partners: “He didn’t want to, busy and because of the work … one might scold him for that. But the baby was already far along, we might not be able to do anything then.” (FGD01, female Thai parent). Though this gender dynamic was not explicitly discussed by Myanmar and Cambodian migrant participants, the patriarchal view of women as subservient to men as heads of households, including in reproductive matters, is pervasive in SEA countries [35].

We also compared migrant and Thai attitudes toward pregnancy termination, given Thailand’s existing thalassemia prevention strategy focused on PNS/PND. All participants agreed that abortion is generally viewed negatively in predominantly Buddhist Thai, Myanmar, and Cambodian cultures. Nearly all participants directly referenced Buddhist teachings of abortion being equivalent to killing a living being and therefore sinful. Many also ascribed their negative view of abortion to a spiritual or moral belief that a fetus wants to live and deserves the chance to “see the world”. A few participants believed that terminating a pregnancy would be a “bad omen” and that “the aborted baby would keep cursing” or haunting them.

Our KAP survey identified a more negative opinion of pregnancy termination among migrants compared to Thai [23]. Indeed, several participants described migrants from Myanmar as being the most conservative, religious, and least educated of the three nationalities – thus the least likely to terminate a pregnancy. However, Cambodian participants viewed their culture as generally more accepting of abortion and reported that medically induced abortions and use of contraception are fairly commonplace in Cambodia. One Cambodian migrant (a Buddhist monk) summarized this more nuanced view of abortion: “Abortion might be against religious teachings … but it’s more about well-being, which is separated from religious beliefs” (A09, Cambodian male agent, 35yo).

Both migrants and Thai also associated stigma and shame with abortion. Several participants voiced disapproval of those who decided to terminate a pregnancy, stating that if they did not want to have a child, they should have used contraceptive methods or been aware of the consequences. As a result, participants reported that abortions were typically performed in secret, in “hidden shops that sell drugs that induce abortion” or using herbal medicine or shamans to induce abortion. Participants also acknowledged the influence of generational hierarchy on attitudes and decisions to terminate; older generations, such as grandparents, were viewed as more conservative and likely to prohibit abortion on behalf of younger generations.

Participants cited similar circumstances leading to abortion, including having insufficient social support from partners or families, not being economically or psychologically ready to have children, not desiring pregnancy at that time, or wanting to focus on work or education. Economic considerations were more commonly cited by Cambodian compared to Thai and Myanmar participants. Factors influencing participants’ willingness to accept pregnancy termination included the viability of the pregnancy, the anticipated severity of the disease leading to future suffering for the child and to high caretaker burden, the availability of effective treatment or cure, gestational age (with abortions being less acceptable beyond the first trimester), and risk of maternal complications from abortion.

Despite nearly universal negative attitudes toward abortion, participants agreed that thalassemia disease should be prevented. In fact, many participants described raising children with severe thalassemia as an unjustified financial, physical, and emotional burden and blamed parents for putting their children and themselves through a shameful experience by not choosing to terminate the pregnancy. However, providers felt that migrants may be more deferential and “obedient” than Thai when it comes to accepting testing or procedures recommended by medical providers. One provider raised the possibility that language barriers and lack of comprehension might translate into perceived deference to authority: “I think that [migrants] trust us enough even when we say they’d risk losing the baby with the [PND] procedures. Or maybe they would listen but not understand us well” (P07, female provider, 40yo).

### Structural and systemic factors

#### Economic considerations influence migrants’ participation in thalassemia prevention

Participant narratives revealed that migrants’ economic concerns are likely to affect the feasibility and acceptability of thalassemia screening. For example, participants stated that the cost of thalassemia screening would be a significant deterrent for migrants if not covered by the government or by health insurance. The inverse was also suggested: one migrant agent felt that migrants would be eager to take advantage of any free healthcare offered regardless of their understanding of thalassemia: “Wherever there are free screenings, we would go there” (A02, Cambodian male migrant, 39yo). In addition, both migrants and providers felt that the financial burden of caring for children with thalassemia may drive migrants to support thalassemia prevention.

Migrant participants also expressed concerns about missing work and often losing a day of wages due to visiting hospitals with long wait times and queues. Many participants stressed that migrants immigrate to Thailand for employment, so work is the foremost priority in their lives. Participants felt that if migrant workers “have to leave work to do the screening, that would be inconvenient” (A03, Myanmar male agent, 30yo), and that migrants would be more likely to engage in thalassemia screening if the testing could be performed rapidly (e.g. using point-of-care testing) in a convenient location.

Despite viewing work as a competing priority, participants felt that employers would be instrumental in facilitating migrants’ access to healthcare, as they are responsible for arranging transportation to the hospital for migrant workers who are sick or need testing. Larger employers often also provide migrant agents to accompany groups of migrant workers to health appointments as interpreters and patient navigators, particularly for the health registration process. Conversely, a handful of providers expressed concern about discrimination, with one Thai provider stating that some employers require migrant workers to report congenital diseases in their paperwork, serving as a basis for discrimination, while another speculated that employers may not want to hire workers with thalassemia because they would “have to visit the hospital regularly and … leave the job quite often,” (P06, female provider, 38yo).

#### Language barriers to healthcare access

In addition to affecting patient-provider communication and education, migrants and providers alike described language barriers as shaping migrants’ experiences of healthcare. Participants gave examples of migrants having difficulties filling out intake forms and reading prescribed medication labels and signs in the hospital written in Thai. Migrant agents described the emotional impact of language barriers on migrant patients: “If they have … someone [who] can speak Thai, it’s good for them. If not, [they] just cry in the room, just cry” (A08, Cambodian male agent, 36yo). Providers also expressed frustration at the limited and inconsistent availability of interpreters in the hospital, who are often either volunteers from non-profit organizations or family members.

#### Legal barriers to healthcare access

Participants described legal barriers faced by migrants, particularly those without documentation, resulting in reluctance to seek health care: “They would be afraid of being asked by the nurses for their passport and, while they’re on their way to the hospital … being apprehended by the police” (A06, Cambodian male migrant, 26yo). Many migrants confirmed this fear with anecdotes of family and friends who were “detained for a week or half a month and then … sent back” to their home country (M07, Cambodian male migrant, 24yo). One provider noted that this fear often led pregnant migrants to present to ANC in later trimesters or to not present until labor. Providers confirmed that migrants were indeed required to present legal documentation during hospital intake and described past instances in which staff called immigration police, but they insisted that hospital staff no longer reported undocumented migrants regularly. In contrast, providers suggested that documentation was secondary to migrants’ ability to pay, and that the hospital would provide any treatment that migrants could afford.

#### Thai views of migrant rights to healthcare and thalassemia screening

Several Thai participants perceived migrants as immigrating into Thailand in large groups, intermarrying with Thai, having many children, and presenting a burden to the already overburdened Thai healthcare system. These perceptions influenced Thai participants’ views on migrants’ rights to thalassemia screening and healthcare in general. For instance, some providers believed that migrants should only be allowed to work but not to have children in Thailand. One provider suggested that thalassemia education should not be offered to migrants by ANC providers if “the real goal is that we don’t encourage them to get pregnant” (FGD04, providers). Another provider felt that contraceptive therapy should be offered to migrants instead of ANC or delivery services “so that we can control the expense” (FGD04, providers).

Thai participants suggested a variety of thalassemia screening approaches in the setting of limited public resources. Some suggested screening migrants selectively, i.e. only in cases of clinical suspicion, or allocating only leftover public funds to migrant screening after universal thalassemia screening for Thai citizens is ensured. Others proposed screening all migrants prior to entry into Thailand and not allowing thalassemia carriers to immigrate in order to reduce the disease burden. Yet others felt that providing migrants with full access to thalassemia screening and preventative healthcare through universal health coverage would reduce migrants’ burden on the healthcare system by decreasing the incidence of thalassemia and other chronic diseases, bolster Thailand’s industrial labor force, or recognize migrants’ inherent rights to healthcare as individuals residing in Thailand.

Notably, a minority of providers felt that migrants should have equal healthcare rights compared to Thai citizens, though nearly all providers reported providing equitable care to migrants. Migrant narratives were mixed; some felt that providers largely “treat everyone equally no matter what” (A09, Cambodian male agent, 35yo), while several migrants recounted experiences in which providers used rude or discriminatory language. Some migrants felt that providers’ attitudes translated into inadequate individual attention, longer wait times, rudimentary medical histories or examinations, or insufficient medications prescribed. These attitudes and behaviors were perceived by migrants to be related to their ability to pay for services, which did impact quality of care according to some providers. For instance, when a specialized procedure is not available at a local hospital, providers and migrants alike may be hesitant to transfer care to a bigger hospital due to migrants’ inability to afford the procedure, leading to suboptimal care.

### Recommended approaches to thalassemia prevention and control in migrant populations

Participants were asked to provide feedback and recommendations for optimal strategies and settings for thalassemia screening. Most participants felt that ANC provided an ideal setting for thalassemia education and screening. Some providers felt that migrants were more compliant with ANC than Thai patients, either because they cared deeply about their children, they desired free healthcare services, or out of a deference to authority. In general, providers saw ANC as an opportunity to engage migrants who might otherwise not seek out healthcare, a narrative confirmed by several migrants who had only sought perinatal care in a formal hospital setting.

Migrants’ perceived desire to have children and unwillingness to terminate a pregnancy were viewed by many participants as a major limitation of the PNS/PND approach. Migrants were thought to hold a traditional belief that having more children is economically advantageous because children will help them work, whereas by contrast, Thai participants felt that “modern” Thai society has “advanced” by utilizing reproductive planning and having fewer children. Migrant narratives provided a mixed view of whether their desire to have children was a factor in their attitudes toward abortion. Providers also expressed concern that migrants may present too late to ANC, eliminating the option for PND and pregnancy termination. In addition, while nearly all migrants expressed interest in being screened during pregnancy, few were aware of the risks and implications of PNS/PND. Providers speculated that migrants would lose interest if they understood that PND carries a risk of spontaneous abortion and may ultimately require termination of affected fetuses.

Some participants suggested that preconception or premarital screening could overcome limitations of PNS/PND and could be done universally for students in schools or universities, couples before marriage, or migrant workers during health registration. Some providers, however, felt that universal carrier screening would be “a waste of money” due to migrants’ transience and poor ability to follow-up on test results. They also questioned whether migrants would change their reproductive behaviors after being diagnosed with thalassemia trait due to poor health literacy and potential indifference about a diagnosis without physical consequences for themselves.

Finally, in terms of the optimal locations for thalassemia screening, some participants felt that the hospital was the most convenient setting for screening because migrants already present there for blood tests and health registration. Furthermore, if an individual is diagnosed and requires treatment, they can be treated on-site. Others felt that going to the hospital for testing created insurmountable structural and economic barriers for migrant workers, and that migrant workplaces were a convenient location for thalassemia screening, allowing migrants to save time and avoid missing work. Some suggested that thalassemia screening be incorporated into annual required employee health checks in order to maximize uptake and that employers be held responsible for educating and screening their employees. However, participants debated whether employers would be willing to take on this role, as on-site testing may be disruptive in some work environments (e.g. construction sites) and as a thalassemia trait diagnosis would not likely affect migrants’ ability to perform their work duties. Furthermore, some expressed concern over the possible loss of confidentiality and workplace discrimination if the screening is done through employers. A proposed alternative setting was the nearby work camps where migrant workers live, which may maximize convenience for workers while protecting their confidentiality. Overall, participants felt that a successful thalassemia prevention program for migrants should focus on providing free and convenient testing services.

## Discussion

This is the first study of barriers and facilitators of thalassemia screening in migrant populations in Thailand. In-depth interviews and FGDs performed with various key stakeholders elucidated educational, attitudinal, socioeconomic, and structural obstacles for implementing a thalassemia prevention and control program for migrants.

Participant narratives confirmed previous KAP survey findings that migrants have very limited awareness and knowledge of thalassemia and genetic disease. Systematic programs utilizing thalassemia education through mass media and carrier screening have led to dramatic reductions in incidence of β-thalassemia in Mediterranean countries [11, 24]. However, our study reveals that migrants’ ability to access mainstream methods of thalassemia education through ANC, public outreach, and mass media are limited by language barriers, health literacy, healthcare providers’ perceptions and cultural competence, and many systemic and legal barriers faced by migrants throughout SEA [27, 29]. Key recommendations from this study include tailoring thalassemia education to migrants’ needs by providing both verbal and written media in migrants’ native languages, providing genetic education and counseling to bolster health literacy, and finding alternative venues for education and outreach outside of ANC to promote access. At the same time, interpreter services and education on cultural competency should be provided to healthcare workers as a critical component of improving patient-provider communication [29].

In line with prior literature using the Health Belief Model (HBM) as a framework for understanding patients’ thalassemia preventive behaviors, [36, 37] our findings suggest that migrants’ knowledge of thalassemia and interest in thalassemia screening are related to their perceptions of personal susceptibility and thalassemia severity. Migrants expressed indifference about screening for a carrier condition that would not affect their physical health or ability to work, especially in light of competing socioeconomic priorities. Misconceptions about thalassemia as a sexually-transmitted disease led some migrants to misjudge their risk of being a thalassemia carrier. Importantly, participant narratives revealed that migrants’ lack of understanding about thalassemia may lead to deference to medical authorities and acceptance of any interventions offered, raising the question of how best to promote thalassemia screening in migrant populations while ensuring individual autonomy. One HBM-based educational program demonstrated that a combination of lectures, group discussions, and pamphlet distribution can successfully increase perceptions of susceptibility, severity, and benefits of screening, improve self-efficacy, and encourage thalassemia testing among participants [37].

Participants also reported that experiential knowledge through friends or family members affected by thalassemia led to more positive attitudes and behaviors toward thalassemia screening and prevention. Therefore, use of peer-to-peer education, outreach by community health workers, patient stories, and audiovisual aids to illustrate key concepts about thalassemia are likely to help promote screening uptake. On the other hand, participant narratives demonstrated that misinformation spread through informal avenues of education and word-of-mouth can lead to the stigmatization of thalassemia carriers [25]. Therefore, creating a centralized educational program that oversees various community outreach approaches will be critical.

Several sociocultural factors are likely to influence migrants’ uptake of thalassemia screening. Despite universally negative perceptions of abortion due to religious and moral beliefs, decisions to terminate a pregnancy affected by thalassemia were described as complex and dependent on other factors, such as the expected severity of the condition, the availability of social support and economic resources, and the couple’s desire for more children. Indeed, prior studies have suggested that despite a central role of religious belief, a spectrum of attitudes and preferences exists surrounding pregnancy termination for severe thalassemia, especially in multicultural societies [25, 38, 39]. Additionally, the prenatal approach relies on the male partner to present for PNS, which may be deprioritized by both male and female partners due to the gender stereotype that men are too busy with work to engage in ANC, as well as hindered by migrants’ limited access to transportation and inflexibility of work schedules.

In light of limitations of the prenatal approach, many participants favored universal carrier screening as a more acceptable approach for migrant communities. However, no consensus appeared on the optimal setting for carrier screening. Migrant workplaces are convenient screening sites but rely on employer engagement and raise concerns about confidentiality. A hospital-based carrier screening program would face similar structural and economic barriers as ANC and PNS/PND. School-based preconception or premarital screening approaches have been successful in a number of countries [40–42] but are less applicable to migrant populations who may not attend school or go through the legal marriage process in Thailand. In addition, carrier screening would require migrants to understand their carrier status, inform their doctors and partners, and modify their reproductive behavior accordingly, demonstrating the importance of first raising thalassemia awareness and health literacy in migrant communities.

Finally, thalassemia prevention efforts are likely to be hindered by migrants’ lack of documentation and access to healthcare. Officially reported numbers of migrants residing in Thailand are likely underestimates due to high volumes of irregular migration, [6] which creates additional barriers, such as fear of arrest and deportation when presenting for healthcare services and inability to register for health insurance [28]. Participant narratives portrayed the Thai healthcare system and especially local hospitals as underequipped and under resourced to handle migrant healthcare needs due to existing high patient volumes and lack of space, interpreter services, and cultural competency. Providers’ narratives also raised concerns about overutilization of resources by migrants, which may hinder the adoption of more culturally competent policies [29]. Our findings emphasize the need to engage policymakers and hospital leadership on the issues of migrant healthcare and reproductive rights. Future studies of the cost-effectiveness of various approaches to thalassemia prevention and control in migrant populations will be necessary to help promote policy decision-making that is both more inclusive of migrants and successful on a national and local hospital level.

### Limitations

The main limitation faced by our study team was the identification of migrants who were well-informed about thalassemia. Most migrants with deep personal experience with thalassemia were unable or unwilling to be interviewed due to work obligations, lack of transportation, or fear of reprisal from employers. Some interviews with migrant workers were hindered by language barriers. As a result, migrant workers’ perceptions of thalassemia were largely gleaned from the experience of providers and agents who had interacted with migrants undergoing ANC. Furthermore, all participants resided in a heavily industrialized province of Thailand with a large migrant population and were associated with a public community hospital. Therefore, participant experiences may not be reflective of those living in less industrial settings or those associated with private hospitals. Finally, only Myanmar and Cambodian migrants were recruited due to language constraints, but most barriers and facilitators identified likely apply to other migrant groups residing in Thailand. Thus, the challenges faced in conducting this study directly reflect the structural barriers that need to be addressed to implement a successful thalassemia prevention and control program for migrants.

## Conclusion

This study highlights key barriers and facilitators that will likely influence the feasibility and acceptability of thalassemia prevention and control measures in SEA migrant populations. We identified several approaches for adapting Thailand’s national thalassemia prevention program to migrant communities, including providing thalassemia education tailored to migrant audiences in both clinical and community settings, adopting carrier screening as part of a prevention strategy, and eliminating structural barriers by providing universal coverage for screening and preventative care for migrants, engaging employers and conducting thalassemia screening in convenient locations, increasing the availability of interpreters in medical facilities, and providing healthcare workers with cultural competency training. With increasing intra-regional migration in SEA, the development of thalassemia prevention and control programs that are inclusive of migrants is urgently needed.

## Data Availability

The datasets generated and/or analyzed during the current study are available from the corresponding authors on reasonable request.

## Abbreviations

FGDs: focus group discussions
SEA: Southeast Asian
PNS: prenatal screening
PND: prenatal diagnosis
ANC: antenatal care
KAP: Knowledge, Attitudes, and Practices
LCH: Laem Chabang Hospital
HBM: Health Belief Model
